# Proceedings of the Royal Society

**Published:** 1872-03

**Authors:** 


*Proceedings of the Royal Society*, London.

We have received from the printers and publishers, Messrs. Taylor and Francis, London, through Mr. Wm. Wesley, Agency of the Smithsonian Institute, 28 Essex Street, Strand, London, No. 123 to 129 inclusive, of the Transactions of the Royal Society.

The proceedings contain essays and papers of much scientific interest, and will form a valuable collection of scientific papers, serving to show the searching interest in matters of science manifested by our brethren across the water. The Royal Society has long been known as one of the most distinguished learned Societies of the world, and their proceedings will be read with interest by all scientific men.

